# Pregnancy Weight Gain and Longer-Term Maternal Cardiometabolic Conditions

**DOI:** 10.1161/HYPERTENSIONAHA.125.26320

**Published:** 2026-06-03

**Authors:** Thais Rangel Bousquet Carrilho, Sonia M. Grandi, Lisa M. Bodnar, Jennifer A. Hutcheon, Kari Johansson

**Affiliations:** Department of Obstetrics and Gynaecology, Faculty of Medicine, University of British Columbia, Vancouver, Canada (T.R.B.C., J.A.H.).; Child Health Evaluative Sciences Program, The Hospital for Sick Children, Toronto, Ontario, Canada (S.M.G.).; Division of Epidemiology, Dalla Lana School of Public Health, University of Toronto, Ontario, Canada (S.M.G.).; Department of Epidemiology, School of Public Health, University of Pittsburgh, PA (L.M.B.).; Division of Clinical Epidemiology, Department of Medicine Solna, Karolinska Institutet, Stockholm, Sweden (K.J.).; Division of Obstetrics, Department of Women’s Health, Karolinska University Hospital, Stockholm, Sweden (K.J.).

**Keywords:** body mass index, cardiovascular diseases, gestational weight gain, hypertension, pregnancy

## Abstract

**BACKGROUND:**

We aimed to establish how weight gain patterns across successive pregnancies relate to longer-term maternal cardiometabolic health.

**METHODS:**

Obstetric records of all nulliparous pregnancies in Stockholm and Gotland (Sweden, 2008–2015) were linked with hospital discharges, outpatient visits, and prescription dispensations until 2019. Total pregnancy weight gain (kg) was standardized for gestational age and early pregnancy body mass index and classified as ≤ −1, > −1 and < +1 (reference), and ≥ +1 *Z* scores. Postpartum cardiometabolic conditions (type 2 diabetes, hypertension, cardiovascular diseases) were identified using *International Classification of Diseases*, *Tenth Revision* codes and medications. Hazard ratios were estimated using a Cox proportional hazards model.

**RESULTS:**

Among 58 333 individuals, 5.9% (n=3440) developed a cardiometabolic condition, with a median onset of 4 years [interquartile range, 2–6]. Hazard ratios were higher for those gaining ≥ +1 *Z* score (19.4 kg at 40 weeks in normal-weight individuals) in the first pregnancy (hazard ratio, 1.29 [95% CI, 1.19–1.40]). Among individuals developing conditions after the second pregnancy, risks were increased for those with high weight gain in the first pregnancy, but not the second (hazard ratios, 1.30 [95% CI, 1.13–1.50] versus 1.05 [95% CI, 0.88–1.26], respectively), compared with those gaining > −1 and < +1 *Z* score in both pregnancies.

**CONCLUSIONS:**

Individuals with high weight gain in their first pregnancy were 30% more likely to develop a cardiometabolic condition than those with lower weight gain. Preventing excessive weight gain in the first pregnancy may be key to reducing maternal cardiometabolic risk.

**Novelty and Relevance**

**What Is New?**

Individuals with a higher weight gain in their first pregnancy had a 30% increased rate of maternal cardiometabolic conditions up to 11 years postpartum.

The rates of cardiometabolic conditions were increased among those who gained more weight in their first but not their second pregnancy.

**What Is Relevant?**

Hypertension was the most common cardiometabolic condition, accounting for 75% of the outcome.

Counseling on the risks of gaining too much weight during pregnancy may be an important target for reducing rates of this condition among nulliparous individuals.

**Clinical/Pathophysiological Implications?**

Obtaining a history of weight gain during pregnancy may help to better understand the risk profile for cardiometabolic conditions in parous individuals.

High pregnancy weight gain has been linked with maternal complications during pregnancy such as gestational diabetes and preeclampsia,^1–3^ but the consequences of excess weight gain for longer-term maternal health are less well understood.^4^ Current pregnancy weight gain guidelines issued by the US Institute of Medicine (IOM) were derived primarily to optimize shorter-term maternal health, due to insufficient evidence on the longer-term health consequences of weight gain for the mother.^4^ The IOM committee called for studies on the association between weight gain and glucose intolerance, hypertension, and cardiovascular risk factors later in a mother’s life.^4^ While some studies have been conducted to fill this gap,^5–11^ they have examined intermediary outcomes or risk scores rather than cardiometabolic conditions themselves^5,6,8,9,11^ or had high loss to follow-up.^5,7–9^ More importantly, studies to date have examined weight gain in each of an individual’s pregnancies separately and have not considered any cumulative effects across successive pregnancies.^5,6,8,10,11^ This is important because most women have more than one pregnancy over the course of their reproductive years.^12^ Thus, the cumulative consequences of weight gain across successive pregnancies on cardiometabolic health remain poorly understood.

An accurate understanding of the relationship between pregnancy weight gain and cardiometabolic conditions could identify a potentially modifiable risk factor for improving longer-term maternal cardiovascular health. As pregnancy is a time when individuals are motivated to make positive behavioral changes,^13^ preventing excess weight gain in pregnancy may be a more effective target for intervention than weight status before or after pregnancy.

In this study, we aimed to establish rates of longer-term maternal cardiometabolic conditions based on weight gain in an individual’s first pregnancy, and by cumulative weight gain patterns across their first 2 pregnancies.

## Methods

### Data Availability

The data included in this study were based on Swedish electronic medical records and national health care registers. According to Swedish law, data sharing is not allowed, and individual-level data in the medical records and registers can only be accessed through secure servers, and only the export of aggregated data is permitted. Data can only be shared for specific collaboration projects between Karolinska Institutet (host) and the collaborating university by signing data processing agreements and a research collaboration agreement. The statistical codes used in the analysis are publicly available at https://osf.io/67hjd.

### Study Population

This was a descriptive longitudinal study following the framework proposed by Lesko et al.^14^ We used data from the Stockholm-Gotland Perinatal Cohort, a population-based cohort of all births ≥20 weeks’ gestation in the Swedish counties of Stockholm and Gotland from 2008 to 2020.^15^ Briefly, the Stockholm-Gotland Perinatal Cohort was created based on prospectively collected, quality-controlled data from electronic medical records of mothers and infants. We identified all nulliparous singleton pregnancies to individuals aged ≥20 years from January 1, 2008, to December 31, 2015, without a prior history of cardiometabolic conditions (cardiovascular disease, renal disease, or prepregnancy diabetes) before the index pregnancy, with a maximum of 3 pregnancies during the study period. For the second pregnancy, we limited to those occurring before January 1, 2016, to ensure a minimum of 4 years of follow-up after both the first and second pregnancies.

We linked pregnancy data with Swedish national health registries from 2008 to 2019. We included hospital discharge summaries and outpatient specialist visits from the National Patient Register,^16^ and prescriptions for drug dispensations from the National Prescribed Drug Register.^17,18^ These data sets were linked using a unique personal identification number assigned to Swedish residents. We also linked the cohort to national mortality from the Cause of Death Register^19^ and immigration from the Total Population Register.^20^ Pregnancies with missing data on early pregnancy body mass index (BMI), missing or implausible weight gain measurements, and individuals who died or emigrated <42 days after delivery were excluded.

### Pregnancy Weight Gain and Early Pregnancy BMI

Total pregnancy weight gain (kg) was standardized for gestational duration into *Z* scores using published Swedish early pregnancy BMI-specific gestational weight gain charts^21^ and classified as ≤ −1, > −1 and < +1, and ≥ +1 *Z* scores. Table 1 describes the values in kg for these cutoffs at 32, 36 and 40 gestational weeks. Because there are no recommended thresholds for weight gain based on the Swedish charts, we used the selected *Z* scores as proxies to approximate clinically meaningful thresholds of insufficient and excessive weight gain.

**Table 1.**
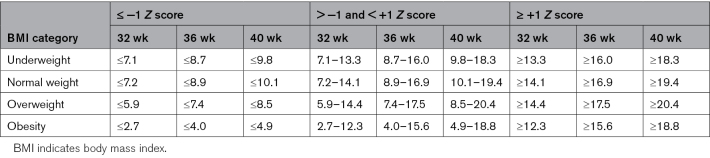
Values of Pregnancy Weight Gain (in kg) at 32, 36, and 40 Weeks for the Selected *Z* Scores, According to the Swedish Pregnancy Weight Gain Charts

Early pregnancy BMI was calculated as the first measured weight <14 weeks’ gestation divided by height in meters squared and categorized as underweight (<18.5 kg/m^2^), normal weight (≥18.5 and <25.0 kg/m^2^), overweight (≥25.0 and < 30.0 kg/m^2^), and obesity (≥30.0 kg/m^2^).^22^

### Cardiometabolic Conditions

Postpartum maternal cardiometabolic conditions were defined as the occurrence of any new-onset diabetes, hypertension, or cardiovascular disease (such as ischemic heart and cerebrovascular disease) >42 days after delivery (either after the first or second pregnancy). Outcomes were identified based on a diagnosis for these conditions using *International Classification of Diseases*, *Tenth Revision* codes,^23^ Swedish version,^24^ available in the National Patient Register,^15^ a prescription for medications used to manage the conditions defined by Anatomic Therapeutic Chemical classification system codes,^25^ Swedish version, available in the National Prescribed Drug Register,^17,18^ or primary or secondary causes of death from the Cause of Death Register.^19^ Codes and algorithms used for case definitions are detailed in Table S1. Outcomes occurring during a third pregnancy or within 42 days of delivery were not considered to avoid inclusion of hypertensive disorders of pregnancy occurring postpartum.

### Statistical Analysis

In this descriptive analysis, we calculated crude risks of cardiometabolic conditions at any time during the follow-up period within categories of weight gain in the first pregnancy. We then used Cox proportional hazards model to estimate hazard ratios (HRs) for cardiometabolic conditions in each weight gain category, adjusting for first-pregnancy baseline characteristics (early pregnancy BMI category, maternal age [years], maternal years of schooling [categorized as <9, 10–12, and >12 years], cohabitation status [living with a partner/other situation], country of birth [Nordic, non-Nordic], and early pregnancy smoking status), with weight gain > −1 and < +1 as the reference category. Because this was a descriptive study, these adjustments were made to account for differences in baseline characteristics and not to isolate causal effects. Cox proportional hazards models were used to account for differences in follow-up. Individuals were followed from 42 days after delivery until the first occurrence of a cardiometabolic condition, date of death, date of emigration from Sweden, date of conception in a third pregnancy, or the end of the follow-up period (December 31, 2019).

Because many individuals have more than one pregnancy during their reproductive years, and risks of cardiometabolic conditions from high pregnancy weight gain may be cumulative across these pregnancies, we then examined the risk of cardiometabolic conditions according to weight gain across a pregnant individual’s first two pregnancies. We were unable to examine third and higher-order pregnancies because of the small number of events. We calculated crude and adjusted HRs for cardiometabolic conditions after the second pregnancy based on a cross-tabulation of weight gain in the first and second pregnancies (with weight gain > −1 and < +1 *Z* score in both pregnancies as the reference). Models were adjusted for the same first-pregnancy variables listed above.

We also calculated crude and adjusted HRs for cardiometabolic conditions according to first-pregnancy weight gain among individuals who experienced a new-onset cardiometabolic condition between their first and second pregnancies (ie, in whom weight gain in the second pregnancy could not have influenced risk of the outcome), and among individuals with only one pregnancy during the study period (and thus had a longer follow-up time available after their first pregnancy). Finally, we estimated HRs of cardiometabolic conditions after a second pregnancy according to the weight gain in the first and second pregnancies independently.

Regression models of cardiometabolic conditions at any time or after the first pregnancy were adjusted for first-pregnancy variables. For cardiometabolic conditions after the second pregnancy, models were adjusted for early pregnancy BMI category, maternal age, maternal education, cohabitation status, country of birth, and smoking status in the second pregnancy.

We conducted a sensitivity analysis in which we further stratified risks of cardiometabolic conditions by first-pregnancy early-pregnancy BMI category. We also compared characteristics and pregnancy outcomes of individuals who did not have a second pregnancy during the study period with those who did, including maternal age, self-reported use of in vitro fertilization, gestational age at delivery, preeclampsia, gestational diabetes, placental abruption (*International Classification of Diseases*, *Tenth Revision* O45),^23^ antepartum hemorrhage (*International Classification of Diseases*, *Tenth Revision* O46),^23^ stillbirth, and infant birth weight.

### Ethical Approval

This study was approved by the Swedish Ethical Review Authority in Stockholm, Sweden (reference number 2009/275-31, amendment 2013/792-32). All clinics in the Stockholm-Gotland Perinatal Cohort database consented to medical record access.

## Results

### Study Participants

Among 218 692 mothers in the Stockholm-Gotland Perinatal Cohort, 197 999 had a valid early pregnancy BMI and pregnancy weight gain measurement. Of these, 58 333 were nulliparous individuals with no history of diabetes or cardiovascular disease, with one or two pregnancies occurring between 2008 and 2015 (Figure S1). Most individuals had normal-weight early pregnancy BMI (70.7%) in their first pregnancy. The median maternal age was 30 years (interquartile range, 27–34), and most individuals had more than 12 years of education (62.5%), lived with a partner (92.1%), were born in a Nordic country (76.0%), and did not smoke (95.4%; Table 2). In addition, 4.4% had preeclampsia and 0.5% had gestational diabetes in the first pregnancy. Of the 58 333 individuals, 27 171 (46.6%) had only 1 pregnancy recorded between 2008 and 2015. Individuals with only 1 pregnancy during the study period had slightly higher rates of use of in vitro fertilization, preeclampsia, gestational diabetes, antepartum hemorrhage, preterm birth, and early pregnancy overweight and obesity than those who had two or more pregnancies (Table S2).

**Table 2.**
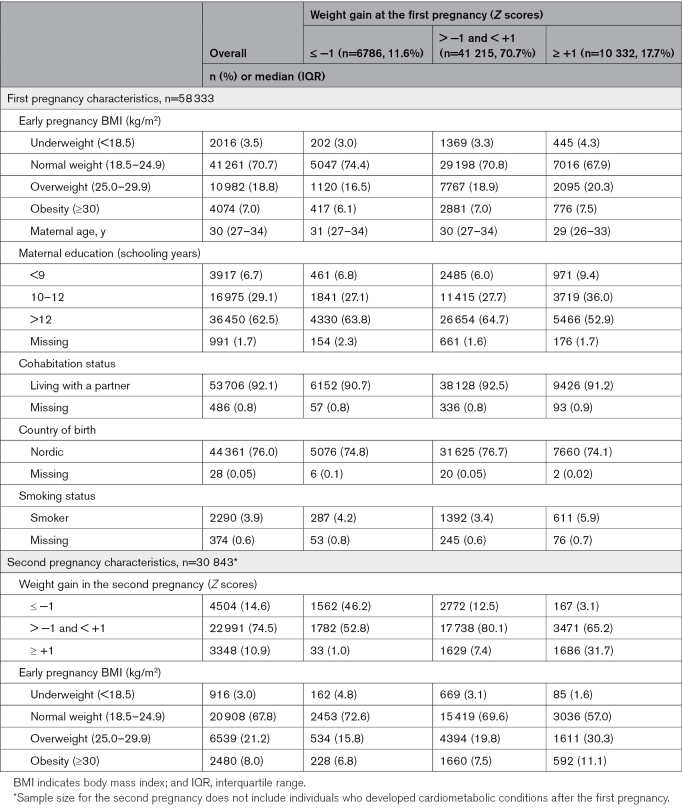
Characteristics of the Study Population According to Weight Gain *Z* Scores in the First Pregnancy (n=58 333)

### Patterns of Pregnancy Weight Gain

Most individuals (70.6%) gained weight > −1 and < +1 *Z* scores in the first pregnancy and remained in the same weight gain category for their two pregnancies. For example, among individuals who gained > −1 and < +1 *Z* score in the first pregnancy, 80.1% also gained > −1 and < +1 *Z* score in the second pregnancy. More individuals gained weight ≥ +1 *Z* score in the first (17.7%) than in the second (10.9%) pregnancy (Table 2).

### Risks of Cardiometabolic Conditions by Pregnancy Weight Gain

The incidence of new-onset postpartum cardiometabolic conditions at any time during the follow-up period was 5.9%, and the median time to onset of cardiometabolic conditions was 4.0 years (interquartile range, 2.0–6.0) after the first pregnancy. Hypertension was the most frequent condition and accounted for ≈75% of the cardiometabolic conditions included. Crude risks of cardiometabolic conditions were higher among those who gained ≥ +1 *Z* score in their first pregnancy (7.1%) compared with individuals who gained > −1 and < +1 *Z* score (5.6%) and individuals who gained ≤ −1 *Z* score (5.8%; Table S3). After adjusting for differences in baseline characteristics, individuals who gained ≥ +1 *Z* score in their first pregnancy had a 30% higher rate of new-onset cardiometabolic conditions (adjusted HR, 1.29 [95% CI, 1.19–1.40]) compared with those who gained > −1 and < +1 *Z* score (Table 3).

**Table 3.**
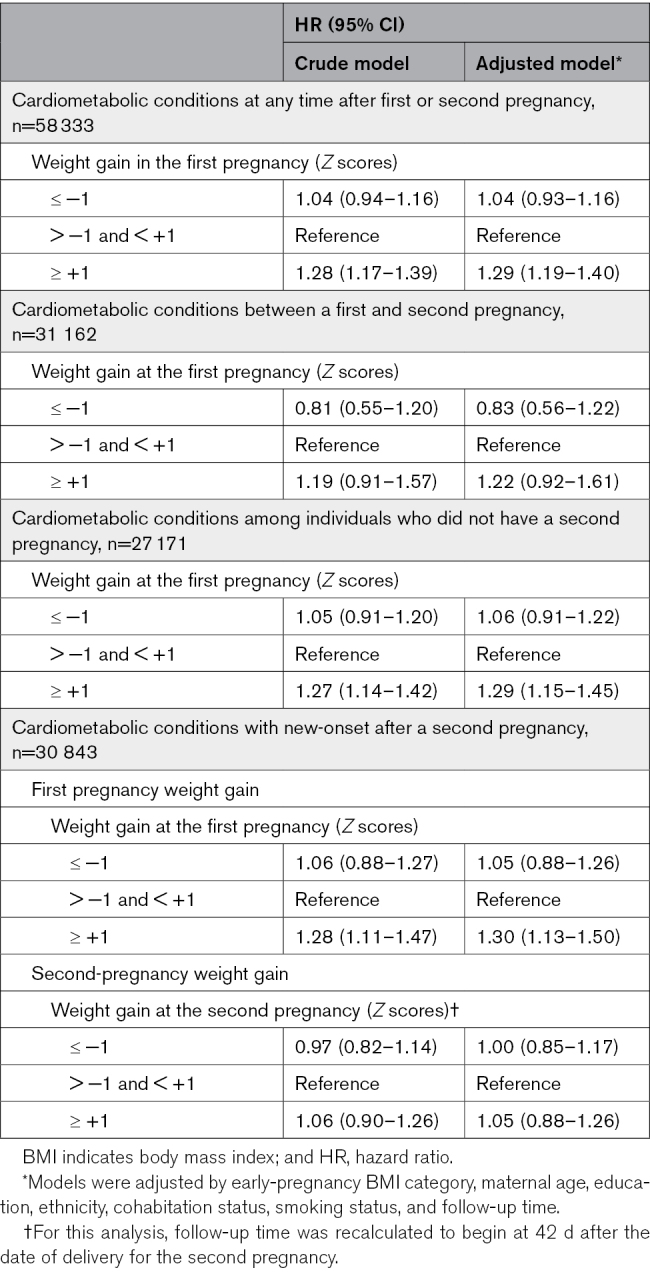
Crude and Adjusted HRs for Cardiometabolic Conditions Within Categories of Weight Gain During Pregnancy

**Figure 1.**
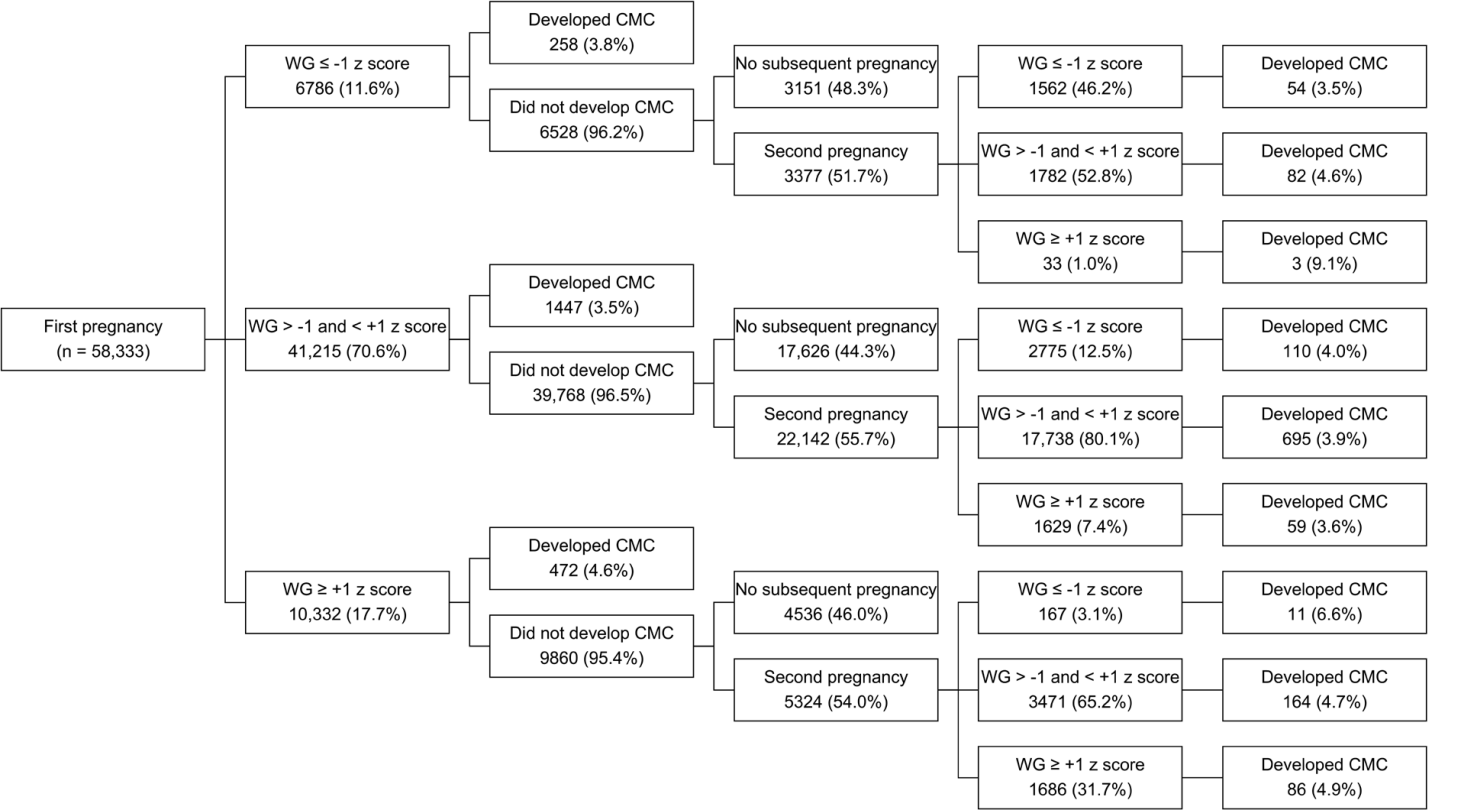
Distribution of cardiometabolic conditions (CMCs) according to weight gain (WG) in the first and second pregnancies.

We then examined rates of cardiometabolic conditions after a second pregnancy according to weight gain patterns across an individual’s first 2 pregnancies and found that weight gain in the first pregnancy was more important than weight gain in the second pregnancy. When we cross-tabulated weight gain categories in the first and second pregnancies, we observed no clear trends between weight gain in the second pregnancy and risk of a subsequent cardiometabolic condition. However, higher crude risks of cardiometabolic conditions were observed among individuals who gained ≥ +1 *Z* score in their first pregnancy, irrespective of their second-pregnancy weight gain category. For example, for individuals who gained ≥ +1 *Z* score in the first pregnancy and ≤ 1 *Z* score in the second, the rate of cardiometabolic conditions was 6.6% (Figure 1).

**Figure 2.**
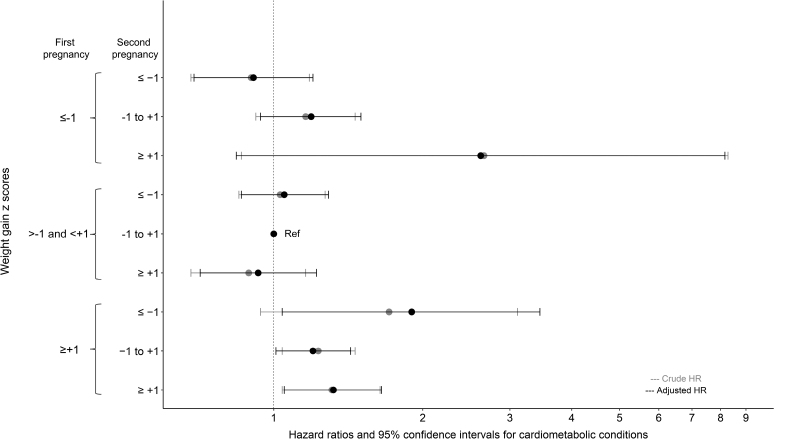
**Crude and adjusted hazard ratios (HRs) and 95% CIs from the Cox proportional hazards model for cardiometabolic conditions after the second pregnancy according to weight gain in the first and second pregnancies.** Models were adjusted by early pregnancy body mass index category, maternal age, education, ethnicity, cohabitation status, smoking status, and follow-up time. Reference group includes individuals who gained > −1 and < +1 *Z* score in both pregnancies.

Among individuals with high first-pregnancy weight gain (≥ +1 *Z* score), adjusted HRs for cardiometabolic conditions after a second pregnancy were increased in all categories of second-pregnancy weight gain (1.90 [95% CI, 1.04–3.45] for ≤ −1 *Z* score, 1.20 [95% CI, 1.01–1.43] for −1 to +1, and 1.32 [95% CI, 1.05–1.65] for ≥ +1) compared with those who gained > −1 and < +1 *Z* score in both pregnancies (Figure 2). Interestingly, high weight gain in both pregnancies was not associated with markedly higher risk levels. For example, individuals with weight gain ≥ +1 *Z* score in two successive pregnancies had similar risks (4.9%) as those with weight gain ≥ +1 *Z* score in their first pregnancy only (4.6%; Figure 1).

Among individuals who experienced a new-onset cardiometabolic condition between their first and second pregnancy, we observed that those who gained ≥ +1 *Z* score in the first pregnancy had higher crude risks (1.2%, Table S3) and an adjusted HR of 1.22 compared with those who gained > −1 and < +1 *Z* score, although the 95% CIs was compatible with a null effect (95% CI, 0.92–1.61; Table 3). For those with only one pregnancy during the study period, we observed crude risks of 8.2% for those who gained ≥ +1 *Z* score in the first pregnancy (Table S3), and a 29% higher HR (adjusted HR, 1.29 [95% CI, 1.15–1.45]) compared with those with weight gain > −1 and < +1 *Z* score (Table 3).

Finally, when we examined the HRs of cardiometabolic conditions after the second pregnancy according to the weight gain in the first and second pregnancies independently, HRs were significantly higher among individuals who gained ≥ +1 *Z* score (adjusted HR, 1.30 [95% CI, 1.13–1.50]), compared with those who gained > −1 and < +1 *Z* score in their first pregnancy. This however was not found in those who gained ≥ +1 *Z* score in the second pregnancy (adjusted HR, 1.05 [95% CI, 0.88–1.22]; Table 3).

### Sensitivity Analyses

We observed higher rates of cardiometabolic conditions (at any time, and after the first or second pregnancy) among individuals with higher weight gain in the first pregnancy (≥ +1 *Z* score) in all early pregnancy BMI categories, especially among those with obesity (Table S4).

## Discussion

### Main Findings

In this population-based study of 58 333 pregnant individuals with up to 11 years of follow-up, we found that the rate of cardiometabolic conditions was ≈30% higher among individuals who gained ≥ +1 *Z* score in the first pregnancy compared with those who gained between −1 and +1 *Z* score. When examining weight gain patterns across successive pregnancies, we found that increased rates of cardiometabolic conditions were linked with high weight gain in the first, but not the second pregnancy.

### Comparison With the Literature

Previous studies have examined the association between pregnancy weight gain above the IOM 2009 guidelines and cardiometabolic outcomes. A study conducted in Sweden examining 6264 participants in the Swedish CardioPulmonary bioImage Study observed higher odds of poor cardiovascular health over an average follow-up time of 24.5 years among individuals with excessive weight gain (adjusted odds ratio, 1.31 [95% CI, 1.09–1.57]) when compared with those who gained weight within the recommendations.^11^ Fraser et al,^5^ analyzing data from 2356 mothers from the Avon Longitudinal Study of Parents and Children, observed that those who gained more weight than the recommendations had higher mean systolic and diastolic blood pressure at 16 years after pregnancy than mothers who gained weight within the recommendations. In Australia, Mamun et al,^7^ using a prospective follow-up study of 3386 mother-child pairs also observed that mothers who gained excess weight during pregnancy had ≈40% (adjusted odds ratio, 1.42 [95% CI 1.07–1.89]) higher chances of developing diabetes by 21 years postpartum, compared with mothers who gained weight according to the recommendations. Finally, in 2022, Hutchins et al^9^ reported that a history of pregnancy weight gain above the 2009 IOM guidelines was associated with a small increase in the atherosclerotic cardiovascular disease risk score at 10 years of follow-up in a US cohort. However, these studies were relatively small cohorts, raising concerns regarding the generalizability of findings due to selective participation and loss to follow-up. Only one of these studies included a population-based sample with longitudinal follow-up and a comparable sample size to the current study. In a population-based study from Denmark, Kirkegaard et al^10^ found that the risk of postpartum hypertension was not associated with weight gain below, within, or above the IOM 2009 guidelines. Our study confirmed the small cohorts’ findings of increased risk associated with higher pregnancy weight gain in a large population-based cohort and extended these findings to demonstrate that the risk of cardiometabolic conditions after a second pregnancy was only higher among individuals with high weight gain in a first but not a second pregnancy.

Other events during pregnancy have previously been identified as predictors of longer-term maternal cardiometabolic health.^26^ The 30% higher rate of new-onset cardiometabolic conditions among individuals with higher weight gain in the first pregnancy observed in this study are lower in magnitude than those observed among individuals with pregnancy complications such as gestational hypertension, preeclampsia, placental abruption, preterm birth and gestational diabetes.^26^ However, because a larger proportion of individuals (17.7%) gain weight ≥1 *Z* score than have one of the aforementioned pregnancy complications (eg, 9.9% of pregnancies result in preterm birth^27^), moderately increased risks could still be linked with a large burden of postpartum cardiometabolic conditions at the population level.

The biologic mechanisms underlying the relationship between higher weight gain during pregnancy (particularly a first pregnancy) and cardiovascular outcomes later in life are not well established. McClure et al^6^ reported that individuals who gained more weight than recommended during pregnancy had greater abdominal adiposity 4 to 12 years after delivery when compared with those who gained within the recommendations. A meta-analysis of 31 prospective studies found that greater abdominal obesity is associated with increased risks of cardiovascular disease in nonpregnant individuals.^28^ Hedderson et al^29^ have hypothesized that excess fat could predispose pregnant individuals to perinatal insulin resistance and the risk could be extended to the postpartum period.^29^ Thus, the greater abdominal adiposity and fat accumulation in general among individuals with excessive weight gain during pregnancy could, in part, explain the higher rates of postpartum cardiometabolic conditions observed in our study. Thus, we hypothesize that the weight gained during the first pregnancy would exert greater stress on the cardiovascular system, resulting in higher blood pressure, increased insulin resistance, increased fat mass, and poorer renal circulation. This would explain why higher rates of cardiometabolic conditions were observed among individuals with high weight gain in the first, but not the second pregnancy.

### Strengths and Limitations

The use of a population-based pregnancy cohort with detailed obstetric information, including gestational weight gain, for successive pregnancies, including those delivered at a different hospitals, is a strength of our study. The large sample size and longitudinal follow-up allowed us to examine the cumulative consequences of weight gain over successive pregnancies and to compare this association among women with one versus two recorded pregnancies during follow-up. The linkage of our pregnancy cohort with Sweden’s national health registers provided us with dates of diagnosis, prescriptions, migration status, and primary and secondary mortality causes, which allowed us to construct a detailed follow-up with minimal losses for up to 11 years for all cohort members.

Although the administrative data used in our study to identify outcomes have been extensively used for research purposes,^15^ they are prone to measurement error. However, our use of different sources for outcome ascertainment (hospital discharges, outpatient visits, prescribed drugs, and mortality) helped to minimize this bias. Despite our large sample size, we still had insufficient data to conduct analyses stratified by early-pregnancy BMI with reasonable statistical precision, especially for underweight; for this reason, we presented BMI-stratified rates as a sensitivity analysis only. The similarity between estimates before and after adjusting by early-pregnancy BMI reinforces that weight gain during a first pregnancy remains an independent risk factor for longer-term cardiometabolic outcomes, regardless of prepregnancy/early-pregnancy BMI. Finally, although the proportion of individuals with missing early-pregnancy BMI or antenatal care weight gain measurements was relatively modest (12%), we lacked data on their characteristics and therefore cannot exclude the possibility of selection bias.

Although the Swedish population is homogeneous and most people have access to prenatal care, the effect of weight gain on outcomes such as preeclampsia and the incidence of conditions such as hypertension are remarkably similar between settings,^3^ supporting the generalizability of our findings to other populations.

### Perspectives

We observed increased rates of cardiometabolic conditions among individuals who gained more weight in the first pregnancy. Our findings suggest that preventing excessive weight gain in nulliparous pregnancies may be an important target for reducing the risk of maternal cardiovascular and metabolic conditions in postpartum.

## Article Information

### Disclosures

None.

### Supplemental Material

Tables S1–S4

Figure S1

## Supplementary Material


