# Supplementary material for: Pregnancy Weight Gain and Longer-Term Maternal Cardiometabolic Conditions
Source: Hypertension. 2026 Jun 3;83(8):e26320. doi: 10.1161/HYPERTENSIONAHA.125.26320 (PMC13367562; doi:10.1161/HYPERTENSIONAHA.125.26320)
Supplement: Supplementary file 1 [file hyp-83-e26320-s001.docx]

**PREGNANCY WEIGHT GAIN AND LONGER-TERM MATERNAL CARDIOMETABOLIC CONDITIONS**

Thais Rangel Bousquet Carrilho^1^, PhD; Sonia M Grandi^2,3^, PhD, Lisa M Bodnar^4^, PhD; Jennifer A Hutcheon^1*^, PhD; Kari Johansson^5,6*^, PhD

1. Department of Obstetrics and Gynaecology, Faculty of Medicine, University of British Columbia, Vancouver, Canada.

2. Child Health Evaluative Sciences Program, The Hospital for Sick Children, Toronto, ON, Canada

3. Division of Epidemiology, Dalla Lana School of Public Health, University of Toronto, ON, Canada

4. Department of Epidemiology, School of Public Health, University of Pittsburgh, Pittsburgh, the USA.

5. Division of Clinical Epidemiology, Department of Medicine Solna, Karolinska Institutet, Stockholm, Sweden.

6. Division of Obstetrics, Department of Women’s Health, Karolinska University Hospital, Stockholm, Sweden

* JAH and KJ contributed equally as senior authors.

**Short title:** Pregnancy weight gain and cardiometabolic conditions

**Corresponding author:** Thais Rangel Bousquet Carrilho. Department of Obstetrics and Gynaecology, Faculty of Medicine, University of British Columbia. 4500 Oak St C408, Vancouver, BC, V6H3N1, Canada. [thaisrangelnut@gmail.com](mailto:thaisrangelnut@gmail.com); phone: +1 604-875-2000 x-6522

# **ONLINE SUPPLEMENTAL MATERIAL**

## **Table S1**. Outcome definitions for cardiometabolic conditions identified via ICD-10 or ATC-codes.

| **Window** | **ICD-10 codes** | **ATC-codes** | **Algorithm for case definition** |
| --- | --- | --- | --- |
| Postpartum, and not during a subsequent pregnancy  (starting from +42 days) | G46·3 - Brain stem stroke syndrome, G46·4 - Cerebellar stroke syndrome, E11-Type 2 diabetes mellitus, E12 - Malnutrition-related diabetes mellitus, I10-I15 - Hypertensive diseases, I20-I25 - Ischaemic heart diseases, I60-I64 - Cerebrovascular diseases, I69·3 - Sequelae of cerebral infarction, I70-I17·4 - Diseases of arteries, arterioles, and capillaries, I77-I79 - Diseases of arteries, arterioles and capillaries,  O10-O11 - Oedema, proteinuria and hypertensive disorders in pregnancy, childbirth and the puerperium | C02 -Antihypertensives, C03 - Diuretics, C07 - Beta blocking agents,  C08 -Calcium channel blockers, C09 -Agents acting on the renin-angiotensin system, C10 -Lipid modifying agents,  A10A - Insulins and analogues, A10B - Blood glucose lowering drugs, excl. insulins | ≥1 diagnostic code from a Hospital Admission, OR  ≥2 diagnostic codes in the specialist outpatient visit register, OR  ≥1 Drug prescription, OR  ≥1 diagnostic code from the delivery hospitalization |

Notes: ATC: Anatomical Therapeutic Chemical Classification System; ICD: International Classification of Diseases

## **Table S2.** Comparison of individuals who did (n = 31,162) *v.* did not have a second pregnancy during the study period (n = 27,171).

| **First pregnancy characteristics** | **Had a second pregnancy**  **(n = 31,162)** | **Did not have a second pregnancy**  **(n = 27,171)** |
| --- | --- | --- |
|  | Median [IQR] | |
| Maternal age (years) | 30 [27, 32] | 31 [27, 35] |
| Birth weight (g) | 3470 [3160, 3790] | 3450 [3130, 3780] |
| Gestational age at delivery (weeks) | 40.1 [39.1, 41.1] | 40.1 [39.1, 41.1] |
|  |  |  |
|  | n (%) | |
| Conceived through IVF | 1586 (5.1) | 2488 (9.2) |
| Had pre-eclampsia | 1277 (4.1) | 1276 (4.7) |
| Had gestational diabetes | 87 (0.3) | 219 (0.8) |
| Had placental abruption* | 103 (0.3) | 113 (0.4) |
| Had antepartum hemorrhage** | 641 (2.1) | 788 (2.9) |
| Delivered a stillbirth*** | 127 (0.4) | 48 (0.2) |
| Delivered a preterm infant (< 37 weeks) | 1584 (5.1) | 1531 (5.6) |
| Early-pregnancy BMI (kg/m^2^) |  |  |
| Underweight (<18.5) | 1039 (3.3) | 977 (3.6) |
| Normal weight (18.5 to 24.9) | 22,872 (73.4) | 18,389 (67.7) |
| Overweight (25.0 to 29.9) | 5443 (17.5) | 5539 (20.4) |
| Obesity (≥ 30) | 1808 (5.8) | 2266 (8.3) |

Notes: * For placental abruption, ICD-10 codes O45 were considered. **For antepartum hemorrhage, ICD-10 codes O46 were used. ***any type of stillbirth

IQR: Interquartile range

## **Table S3.** Cardiometabolic conditions rates by weight gain z scores in the first pregnancy (n = 58,333).

| ***Cardiometabolic condition rates*** | **Overall** | **Weight gain at the first pregnancy (*z* scores)** | | |
| --- | --- | --- | --- | --- |
|  |  | **≤ -1**  **(n = 9205, 11.7%)** | **> -1 and < +1**  **(n = 55,855, 70.8%)** | **≥ +1**  **(n = 13,847, 17.5%)** |
|  | **n (%)** | | | |
| At any time (after first or second pregnancy) (n = 58,333) | 3440 (5.9) | 393 (5.8) | 2311 (5.6) | 733 (7.1) |
| After a second pregnancy (n = 30,843) | 1263 (4.1) | 138 (4.1) | 864 (3.9) | 261 (4.9) |
| Between a first and second pregnancy (n = 31,162) | 319 (1.0) | 28 (0.8) | 226 (1.0) | 65 (1.2) |
| Among individuals who did not have a second pregnancy (n = 27,171) | 1858 (6.8) | 230 (6.8) | 1121 (6.5) | 407 (8.2) |

## **Table S4.** Cardiometabolic conditions rates by early-pregnancy BMI and weight gain z scores in the first pregnancy (n = 58,333).

|  | **Underweight**  **(n = 2016,**  **3.5%)** | | | **Normal weight**  **(n = 41,261,**  **70.7%)** | | | **Overweight**  **(n = 10,982,**  **18.8%)** | | | **Obesity**  **(n = 4074,**  **7.0%)** | | |
| --- | --- | --- | --- | --- | --- | --- | --- | --- | --- | --- | --- | --- |
|  | Weight gain at the first pregnancy (*z* scores) | | | | | | | | | | | |
| Cardiometabolic condition rates | ≤ -1 | > -1 and < +1 | ≥ +1 | ≤ -1 | > -1 and < +1 | ≥ +1 | ≤ -1 | > -1 and < +1 | ≥ +1 | ≤ -1 | > -1 and < +1 | ≥ +1 |
|  | n (%) | | | | | | | | | | | |
| At any time (after the first or second pregnancy) | 4 (2.0) | 58 (4.2) | 17 (3.8) | 241 (4.8) | 1384 (4.7) | 406 (5.8) | 93 (8.3) | 518 (6.7) | 194 (9.3) | 58 (13.9) | 351 (12.2) | 116 (15.0) |
| After at second pregnancy | 1 (1.0) | 22 (3.1) | 6 (2.6) | 91 (3.5) | 591 (3.6) | 167 (4.4) | 30 (6.2) | 159 (4.1) | 58 (5.6) | 16 (8.4) | 92 (7.3) | 30 (9.7) |
| Between a first and second pregnancy | 0 | 9 (1.3) | 1 (0.4) | 21 (0.8) | 138 (0.8) | 38 (1.0) | 6 (1.2) | 43 (1.1) | 19 (1.8) | 1 (0.5) | 36 (2.8) | 7 (2.2) |
| Among individuals who did not have a second pregnancy | 3 (2.9) | 27 (4.1) | 10 (4.6) | 129 (5.3) | 655 (5.1) | 201 (6.2) | 57 (9.1) | 316 (8.2) | 117 (11.3) | 41 (18.1) | 223 (14.1) | 79 (17.1) |

**
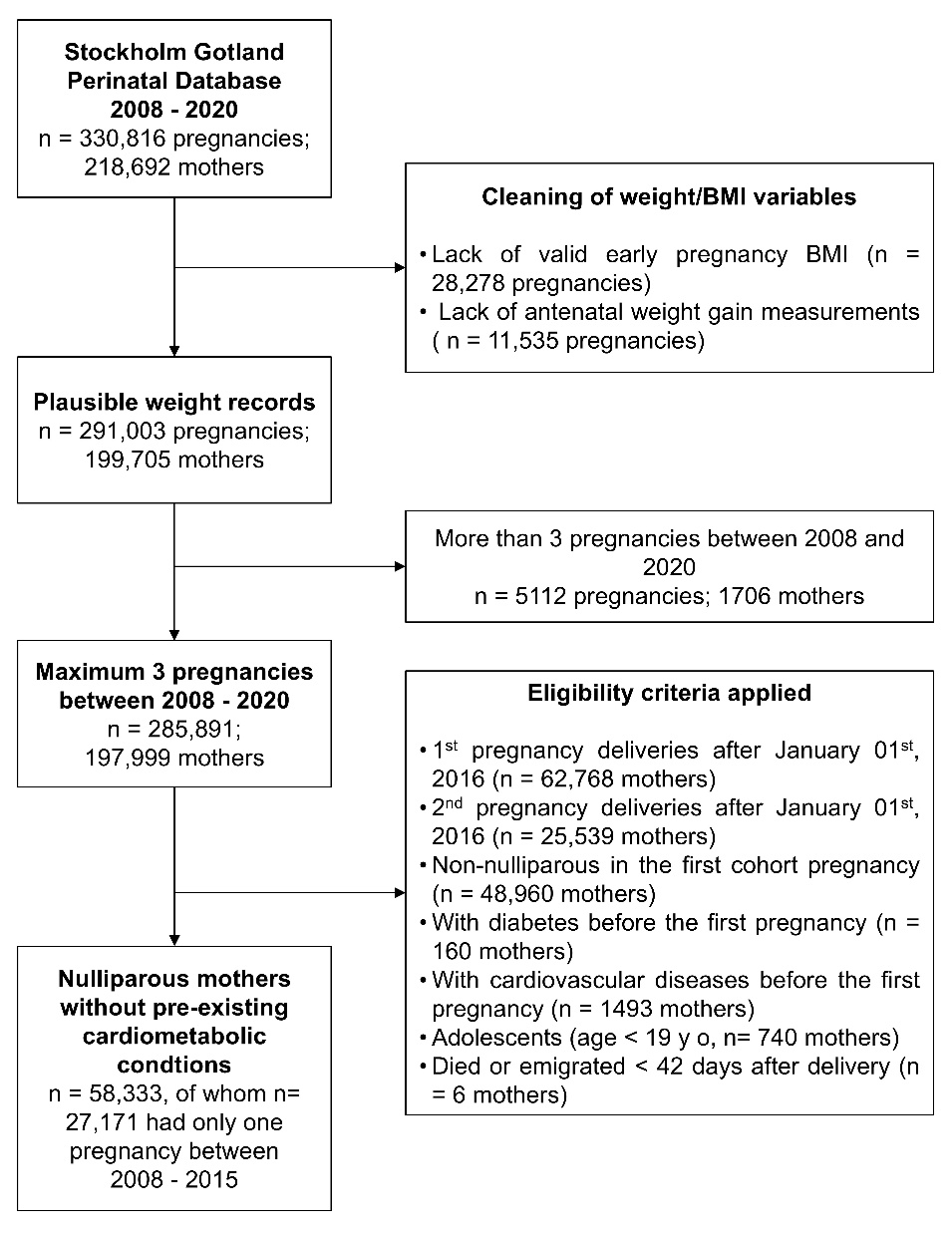
**

## **Figure S1.** Flow of participants delivering from 2008 to 2015 (with follow-up until 2019), Stockholm-Gotland Perinatal Cohort, Sweden.
